# Identification and fine mapping of a major QTL (*qRtsc8-1*) conferring resistance to maize tar spot complex and validation of production markers in breeding lines

**DOI:** 10.1007/s00122-022-04053-8

**Published:** 2022-02-18

**Authors:** Jiaojiao Ren, Penghao Wu, Gordon M. Huestis, Ao Zhang, Jingtao Qu, Yubo Liu, Hongjian Zheng, Amos E. Alakonya, Thanda Dhliwayo, Michael Olsen, Felix San Vicente, Boddupalli M. Prasanna, Jiafa Chen, Xuecai Zhang

**Affiliations:** 1 College of Agronomy, Xinjiang Agricultural University, Urumqi, 830052 China; 2 International Maize and Wheat Improvement Center (CIMMYT), Texcoco, Mexico; 3 College of Bioscience and Biotechnology, Shenyang Agricultural University, Shenyang, Liaoning China; 4 Maize Research Institute, Sichuan Agricultural University, Wenjiang, Sichuan China; 5 CIMMYT-China Specialty Maize Research Center, Crop Breeding and Cultivation Research Institute, Shanghai Academy of Agricultural Sciences, Shanghai, 201403 China; 6 International Maize and Wheat Improvement Center (CIMMYT), Village Market, P. O. Box 1041, Nairobi, 00621 Kenya; 7 College of Life Science, Henan Agricultural University, Zhengzhou, 450002 China

## Abstract

**Key message:**

**A major QTL of** ***qRtsc8-1*** **conferring TSC resistance was identified and fine mapped to a 721 kb region on chromosome 8 at 81 Mb, and production markers were validated in breeding lines.**

**Abstract:**

Tar spot complex (TSC) is a major foliar disease of maize in many Central and Latin American countries and leads to severe yield loss. To dissect the genetic architecture of TSC resistance, a genome-wide association study (GWAS) panel and a bi-parental doubled haploid population were used for GWAS and selective genotyping analysis, respectively. A total of 115 SNPs in bin 8.03 were detected by GWAS and three QTL in bins 6.05, 6.07, and 8.03 were detected by selective genotyping. The major QTL *qRtsc8-1* located in bin 8.03 was detected by both analyses, and it explained 14.97% of the phenotypic variance. To fine map *qRtsc8-1,* the recombinant-derived progeny test was implemented. Recombinations in each generation were backcrossed, and the backcross progenies were genotyped with Kompetitive Allele Specific PCR (KASP) markers and phenotyped for TSC resistance individually. The significant tests for comparing the TSC resistance between the two classes of progenies with and without resistant alleles were used for fine mapping. In BC_5_ generation, *qRtsc8-1* was fine mapped in an interval of  ~ 721 kb flanked by markers of KASP81160138 and KASP81881276. In this interval, the candidate genes *GRMZM2G063511* and *GRMZM2G073884* were identified, which encode an integral membrane protein-like and a leucine-rich repeat receptor-like protein kinase, respectively. Both genes are involved in maize disease resistance responses. Two production markers KASP81160138 and KASP81160155 were verified in 471 breeding lines. This study provides valuable information for cloning the resistance gene, and it will also facilitate the routine implementation of marker-assisted selection in the breeding pipeline for improving TSC resistance.

**Supplementary Information:**

The online version contains supplementary material available at 10.1007/s00122-022-04053-8.

## Introduction

Tar spot complex (TSC) is one of the major foliar diseases of maize in many Central and Latin American countries. The disease can cause greater than 50% grain yield loss in susceptible maize genotypes and reduce fodder quality (Pereyda-Hernández et al. 2009; Loladze et al. 2019; Mottaleb et al. 2019). TSC was first reported in Mexico and most often prevalent in moderately cool and humid tropical and subtropical areas. TSC has for long been associated with at least three fungal pathogens: *Phyllachora maydis*, *Monographella maydis*, and *Coniothyrium phyllachorae* (Hock et al. 1992). *Phyllachora maydis* is the most important pathogen involved in TSC, which alone can cause tar spots and severe losses on maize yield.

Appropriate crop management practices including early or on-time sowing, lower densities, fungicidal control, and use of TSC resistant varieties are the traditional approaches to control TSC. Breeding TSC resistant varieties is the most cost-effective, environmentally friendly, and long-term approach to reduce the economic impact caused by TSC, which depends on the collection and identification of resistant germplasms. In the past 30 years, the International Maize and Wheat Improvement Center (CIMMYT) has developed and distributed many TSC resistant maize varieties (Mottaleb et al. 2019). A large number of lines were identified for TSC resistance under natural disease screening conditions, and some lines showed high levels of resistance to TSC, which are valuable donors for breeding TSC resistant varieties and dissecting the genetic architecture of TSC resistance. Several studies have been conducted to identify quantitative trait loci (QTL) conferring TSC resistance (Mahuku et al. 2016; Cao et al. 2017).

Genome-wide association study (GWAS), linkage mapping, and selective genotyping are powerful methods in genetic research (Sun et al. 2010; Cao et al. 2017; Gowda et al. 2021). GWAS based on linkage disequilibrium (LD) can effectively detect genetic variants associated with the target trait, but it is hampered by high false positive associations (Yuan et al. 2019; Liu et al. 2021). Linkage mapping based on recombination events is a powerful method for QTL mapping of complex traits, but the mapping resolution is low (Cao et al. 2017). Selective genotyping of individuals with extreme phenotypic values from one or both tails is a cost-effective strategy, which may provide roughly equivalent power to complete QTL mapping (Lebowitz et al. 1987; Lee et al. 2014). The combined use of different mapping methods can complement the limitations of each other and has been successfully applied in revealing the genetic basis of several major diseases in maize (Guo et al. 2020; Ren et al. 2021). A major QTL of *qRtsc8-1* on chromosome 8 was detected and verified in different genetic populations by the combined use of GWAS and linkage mapping in previous studies (Mahuku et al. 2016; Cao et al. 2017). Fine mapping of *qRtsc8-1* and development of the production markers that are tightly linked to this major QTL will lead to improvement in the application of marker-assisted selection (MAS) against TSC resistance.

The recombination-derived progeny test strategy is a powerful and widely used method for QTL fine mapping, which can narrow down the genomic region of the target QTL through trait-marker association testing in recombination-derived progenies (Ding et al. 2012; Liu et al. 2016). Using the recombinant-derived progeny test, a QTL *qMrdd8* associated with maize rough dwarf disease resistance was fine mapped to an interval of 347 kb, and two candidate genes CG1 and CG2 were identified (Liu et al. 2016). In addition, a major QTL *RppCML496* conferring resistance to *Puccinia polysora* in maize was fine mapped to an interval of 128 kb and a NBS-LRR gene was the most likely candidate gene (Lv et al. 2021).

The development of functional markers within the finely mapped interval of the major QTL will enable the introgression of resistant alleles into elite breeding material through MAS, after the marker effects are verified in breeding materials. Compared to conventional breeding, MAS breeding is an indirect selection technique, where the selection is based on markers tightly linked to the genomic regions regulating the target trait, rather than the trait itself (Badu-Apraku and Fakorede 2017). Subsequently, the favorable alleles are transferred from the donor lines to the recipient lines for the improvement of the target trait. Several maize breeding programs have reported improved selection efficiency upon implementation of MAS (Nair et al. 2015; Xu et al. 2020; Prasanna et al. 2020a, 2021). At CIMMYT, MAS is being routinely deployed to enrich the favorable alleles of large effect QTL for maize lethal necrosis, maize streak virus (MSV), and provitamin A content in tropical maize breeding populations (Prasanna et al. 2020a, b, 2021).

Fine mapping the major QTL for TSC resistance and verification of the effects of production markers in breeding lines are essential to accelerate the development of TSC resistant germplasm via MAS. Several large effect QTL conferring TSC resistance have been detected, but none of these QTL has been fine mapped and therefore production markers for routinely implementing MAS are still unavailable. The objectives of this study were to (1) identify the major QTL conferring TSC resistance by the combined use of GWAS and selective genotyping, (2) fine map the major QTL of *qRtsc8-1* by subjecting the BC_1_, BC_3_, and BC_5_ progenies to recombination-derived progeny testing, and reveal the candidate genes in the fine mapped interval, (3) develop and validate the production markers in breeding lines for the routine deployment of MAS to enrich the favorable alleles of *qRtsc8-1* in tropical maize breeding populations.

## Materials and methods

### Plant materials

A total of 646 diverse maize inbred lines, including two association mapping panels of Drought Tolerant Maize for Africa (DTMA) and CIMMYT maize lines (CMLs), were used for GWAS. The DTMA panel and the CMLs panel, representing broadly the genetic diversity of tropical/subtropical maize, consisted of 282 and 364 inbred lines, respectively. A bi-parental doubled haploid (DH) population consisting of 201 lines was used for selective genotyping to confirm the genomic region of the major QTL of *qRtsc8-1* identified by GWAS. It was derived from the F_1_ cross formed between the TSC resistant line of CML495 and the TSC susceptible line of La Posta Sequia C7 F64-2-6-2-2-B-B-B.

**Fig. 1:**
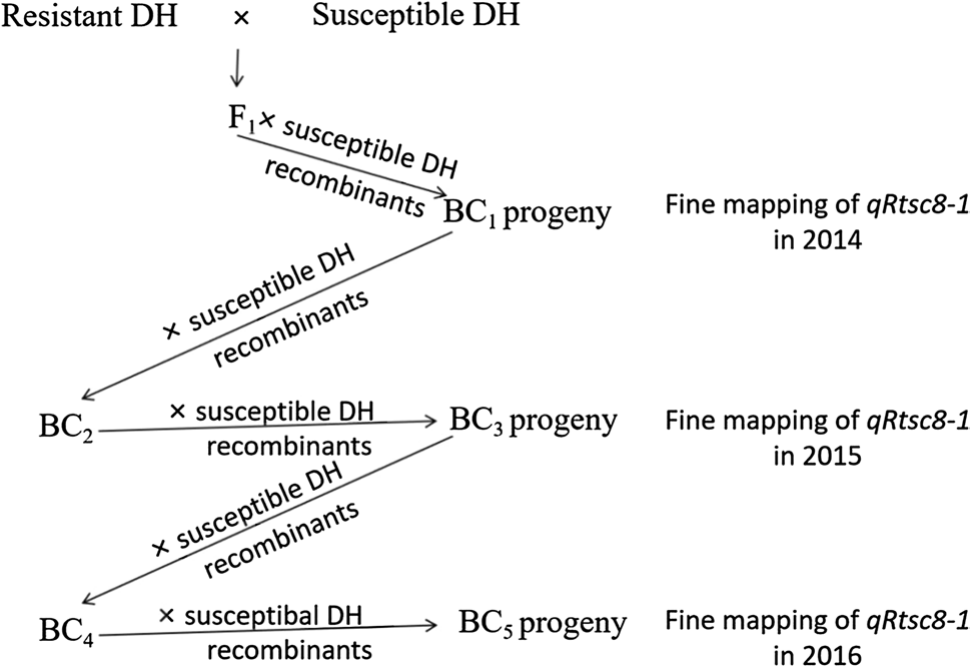
Experimental flowchart for fine mapping of *qRtsc8-1*

Fine mapping was performed in the DH population (Fig. 1). Resistant DH lines were selected and crossed to the susceptible DH lines to generate F_1_ populations. Molecular markers within the *qRtsc8-1* region were used to identify recombinants, which were backcrossed to the recurrent susceptible DH lines to generate the BC_1_ progenies. The BC_1_ progenies were planted to evaluate for resistance to TSC. This process was repeated to develop a series of advanced backcross populations, including BC_2_, BC_3_, BC_4_, and BC_5_ to fine map the QTL of *qRtsc8-1*. A breeding population consisted of 471 breeding lines was used to validate the fine mapping results and verify the effects of production markers. In total, 360 of the 471 breeding lines were overlapped with those in the two GWAS panels.

### Experimental design

All the field experiments were carried out under natural disease screening conditions at CIMMYT’s lowland tropical experimental station in Agua Fria (20°28´N, 97°38´W; 110 masl) in Mexico, a location with high and consistent TSC disease pressure. The DTMA panel was evaluated for resistance to TSC in 2011, 2012, and 2014. The CMLs panel was evaluated in 2017, 2018 with two planting dates, and 2019. The DH population was evaluated in 2012, 2013, and 2014. Of the 471 breeding lines, 395 lines were CMLs which were evaluated for resistance to TSC in 2017, 2018 with two planting dates, and 2019. The rest of the 76 lines were screened for TSC resistance in 2013 and 2016. All the populations were evaluated in a randomized complete block design with three replications. Eleven seeds were sown in a 2 m plot with 0.75 m row spacing.

### Disease evaluations in the field

Disease resistance was evaluated as described by Mahuku et al. (2016) under natural disease screening conditions. Disease severity was conducted three times at weekly intervals, starting from two weeks after flowering. Disease severity was scored using a 1–5 rating scale, where 1 corresponds to highly resistant with no visible disease symptoms and nearly 0% of leaves infected; 2 corresponds to resistant with 1–30% of the leaf area infected; 3 corresponds to moderately susceptible with 31–50% of the leaf area infected; 4 corresponds to susceptible with 51–75% of the leaf area infected; and 5 corresponds to highly susceptible with 76–100% of the leaf area infected. The final highest score was used for further analyses. For initial QTL mapping, disease severity was scored throughout plots. For fine mapping, the severity of each plant was scored individually, and the TSC score within each genotypic class was calculated by the following formula: TSC score = ∑ (severity scale × number of planes per scale)/the total number of plants.

### Phenotypic data analysis

Phenotypic data analysis was performed using META-R Version 6.04 (Alvarado et al. 2020). Best linear unbiased predictions (BLUPs) and variance components were estimated by a mixed linear model (MLM):

$$Y_{ijk} \, = \,\mu \, + \,G_{i} \, + \,E_{j} \, + \,R\left( E \right)_{kj} \, + \,GE_{ij} \, + \,\varepsilon_{ijk} ,$$

where *Y*_*ijk*_ is the phenotypic value of the *i-*th genotype at the *j-*th environment in the *k-*th replication, *μ* is the overall mean, *G*_*i*_ is the effect of the *i*-th genotype, *E*_*j*_ is the effect of the *j-*th environment, * R*(*E*)_*kj*_ is the effect of the *k-*th replication at the *j-*th environment, *GE*_*ij*_ is the effect of *i-*th genotype by *j-*th environment, and *ε*_*ijk*_ is the residual. All the factors were set as random effects. Heritability was calculated on an entry-mean basis as defined by Hallauer et al. (2010).

### Genotyping by sequencing

Young leaves of plants for the GWAS and selective genotyping analysis were sampled for DNA extraction. Genomic DNA was extracted using a CTAB procedure (CIMMYT 2005) and genotyped using the genotyping by sequencing (GBS) platform (Elshire et al. 2011; Wu et al. 2016; Wang et al. 2020) at Cornell University Biotechnology Resource Center (Ithaca, NY). SNP calling was performed according to the method described by Cao et al. (2017). A total of 955,690 SNPs including 570 SNPs with unclear positions were obtained based on Maize B73 RefGen_v2 reference genome. Imputation was performed using the FILLIN method in TASSEL 5.0 software (Bradbury et al. 2007) to reduce the number of missing data.

### GWAS analysis

For the imputed SNP dataset, SNPs were filtered with missing rate < 20%, heterozygosity rate < 5%, and a minor allele frequency > 0.05. A total of 248,482 high-quality SNPs evenly distributed on maize ten chromosomes were retained for future analysis. Analyses of linkage disequilibrium (LD) and GWAS were performed with 646 maize inbred lines in TASSEL 5.0. The LD decay was estimated as the Squared Pearson correlation coefficient (*r*^*2*^) calculated between adjacent SNPs. The threshold *r*^*2*^ = 0.1 was used. The distance of LD decay was 5.04 kb across the ten chromosomes. The LD decay distance ranged from 7.94 kb on chromosome 4 to 1.92 kb on chromosome 6. The MLM incorporating kinship (K) matrix and principal component analysis (PCA) was applied for GWAS analysis. The K matrix was estimated with the default Centered_Identity by State method in TASSEL 5.0. The first three principal components were calculated to control the population structure. The threshold *P* value of 2.01 × 10^–7^ was determined by a Bonferroni correction method to avoid false positives. The GWAS results were used to generate Manhattan and quantile–quantile (q-q) plots with the qqman package in R software (R Core Team 2019).

### Selective genotyping

In the DH population, 20 DH lines with the highest TSC scores (top 10% susceptible tail) and 20 DH lines with the lowest TSC scores (top 10% resistant tail) were selected to detect QTL by selecting genotyping. For the unimputed SNP dataset, 34,317 SNPs with missing rate < 20%, heterozygosity rate < 5%, and a minor allele frequency > 0.10 were used for selective genotyping analysis. A Chi-square test with a 2 × 2 contingency table was used for the comparison of allele frequencies in resistant and susceptible groups. The SNP showing a significant difference (*P* < 0.05) between the allele frequencies of the two tails indicates the presence of a resistance QTL near this SNP. The threshold *P* value (1.46 × 10^–6^) was determined by a Bonferroni correction method. The qqman package in R software was used to visualize the selective genotyping results.

### Development of KASP markers

KASP markers were used to fine mapping *qRtsc8-1*. Sequences of KASP markers whose names start with PZA or PHM were obtained from the maize KASP assays developed for CIMMYT’s Global Maize Program and the Generation Challenge Programme (https://www.biosearchtech.com/products/pcr-kits-and-reagents/genotyping-assays/kasp-genotyping-chemistry/kasp-snp-libraries/maize-genotyping-library). Polymorphic SNPs between CML495 and La Posta Sequia C7 F64-2-6-2-2-B-B-B were selected based on the GBS dataset and used to develop new KASP markers. A total of 19 KASP markers within the physical interval identified by GWAS and selective genotyping were developed and named starting with “KASP” (Table S1).

### Fine mapping strategy of *qRtsc8-1*

A recombinant-derived progeny testing strategy (Yang et al. 2010) was used for fine mapping of *qRtsc8-1* (Fig. 1). Recombinants identified from all mapping populations (F_1_, BC_2_, and BC_4_) were backcrossed to the corresponding susceptible DH lines to produce backcross progenies (BC_1_, BC_3_, and BC_5_). Individuals derived from each recombinant-derived backcross progeny were planted to evaluate for resistance to TSC and genotyped with appropriate markers using KSAP assays (LGC Genomics). They were classified into two classes of genotype in the *qRtsc8-1* region: homozygous La Posta Sequia C7 F64-2-6-2-2-B-B-B and heterozygous La Posta Sequia C7 F64-2-6-2-2-B-B-B/CML495. A two-way ANOVA was used to compare disease scores between the two genotypic classes. A significant difference (*P* < 0.05) between the TSC resistance scores of the two genotypic classes indicated that the resistance QTL was present in the heterozygous region. Otherwise, it indicated that the resistance QTL was absent in the heterozygous region.

### Candidate gene analysis

The B73 sequence of the fine mapping interval was obtained through maizeGDB (Portwood et al. 2019). Candidate genes were retrieved and annotated based on the maize B73 RefGen_v2 reference genome. Meanwhile, the B73 RefGen_v4 reference genome was updated in the supplemental tables to provide more precise information of the significantly associated SNPs and the candidate genes for further research. Genetic variation analysis of candidate genes was conducted using resequencing data of the two parental lines based on the B73 RefGen_v4 reference genome (data not published). Sequences of each gene and 2 kb upstream of the transcription start sites were used to perform genetic variation analysis with the HaplotypeCaller module by Genome Analysis Toolkit (GATK) software (McKenna et al. 2010). The identified variations were further annotated with SnpEff software (Cingolani et al. 2012).

### Haplotype analysis and verification production markers in breeding lines

Ten genotyping assays were developed at LGC genomics for haplotype analysis and verification of the production markers in 471 breeding lines. In total, 8 of the 10 genotyping assays passed the technical validation process in the breeding lines, which were used as the production markers for further haplotype and verification analyses. To determine the functional markers for deploying MAS routinely in breeding populations, a stepwise regression of TSC resistance with the genotype was carried out with the R MASS package. LD and haplotype analysis were conducted by Haploview 4.2 (Barrett et al. 2005). Standardized disequilibrium coefficient (D’) was used to evaluate the LD between markers and generate the LD heatmap. Haploid blocks were detected based on LD using the confidence intervals method in Haploview 4.2 (Gabriel et al. 2002).

## Results

### Evaluation of the resistance to TSC

The descriptive statistics for resistance to TSC in the DTMA panel, CMLs panel, the DH population, and the breeding lines are presented in Table 1. The means of TSC score in all four populations were similar, ranging from 2.30 in the DTMA panel to 2.50 in the DH population. Broad variations were observed in all populations. The TSC scores in the breeding lines showed the largest difference, ranging from 1.15 to 4.04, whereas the DH population showed the smallest difference, ranging from 1.78 to 3.56. Heritability of TSC resistance was 0.72 in the DTMA panel, 0.87 in the CMLs panel, and 0.74 in the DH population. The high heritability indicated that genetics accounted for much of the phenotypic variation in each population.

**Table 1:** Descriptive statistics of response to tar spot complex (TSC) in the Drought Tolerant Maize for Africa (DTMA) panel, CIMMYT maize inbred lines (CMLs) panel, the bi-parental doubled haploid (DH) population, and the 471 breeding lines

Population	Mean	Max	Min	Median	Standard Deviation	Skewness	Kurtosis	Heritability
DTMA	2.30	3.46	1.36	2.28	0.39	0.33	−0.28	0.72
CMLs	2.38	3.67	1.23	2.51	0.49	−0.09	−0.37	0.87
DH	2.50	3.56	1.78	2.47	0.37	0.49	−0.02	0.74
Breeding lines	2.45	4.04	1.15	2.46	0.55	0.09	−0.29	–

### Significant SNPs identified by GWAS

**Fig. 2:**
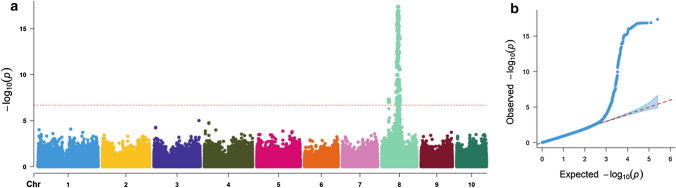
Manhattan and quantile–quantile (q−q) plots of genome-wide association study (GWAS) results for tar spot complex (TSC) resistance using all the 646 diverse lines from both DTMA panel and CMLs panel. **a** Manhattan plot, the horizontal line represents the threshold *P* value = 2.01 × 10^–7^; **b** q−q plot

The GWAS results are shown in Fig. 2 and Table S2. GWAS revealed that 115 SNPs were significantly associated with TSC resistance. All SNPs were located in bin 8.03 but distributed in two genomic regions. Two SNPs of S8_35003118 (*P* = 5.22 × 10^–8^) and S8_35003139 (*P* = 9.22 × 10^–8^) were located in the same genomic region, and the remaining 113 SNPs were located in another genomic region between 73,734,307 bp to 90,835,374 bp with an interval of 17.10 million base pairs (Mb) on chromosome 8 (B73 RefGen_v2). These significantly associated SNPs individually explained 5.21–14.97% of the total phenotypic variance. The most significant SNP S8_81160155 was identified at position 81,160,155 bp on chromosome 8 with the smallest *P* value of 4.36 × 10^–18^, which explained 13.57% of phenotypic variance. The q-q plot showed that population structure was well controlled using the MLM (PCA + K) method in TASSEL 5.0. The statistical model (MLM) and *P* value threshold (2.01 × 10^–7^) used in the present study are strict, which could avoid false positives, but it may lead to the loss of power to detect some significantly associated SNPs on other chromosomes, rather than only on chromosome 8.

### QTL detected by selective genotyping for TSC resistance

**Fig. 3:**
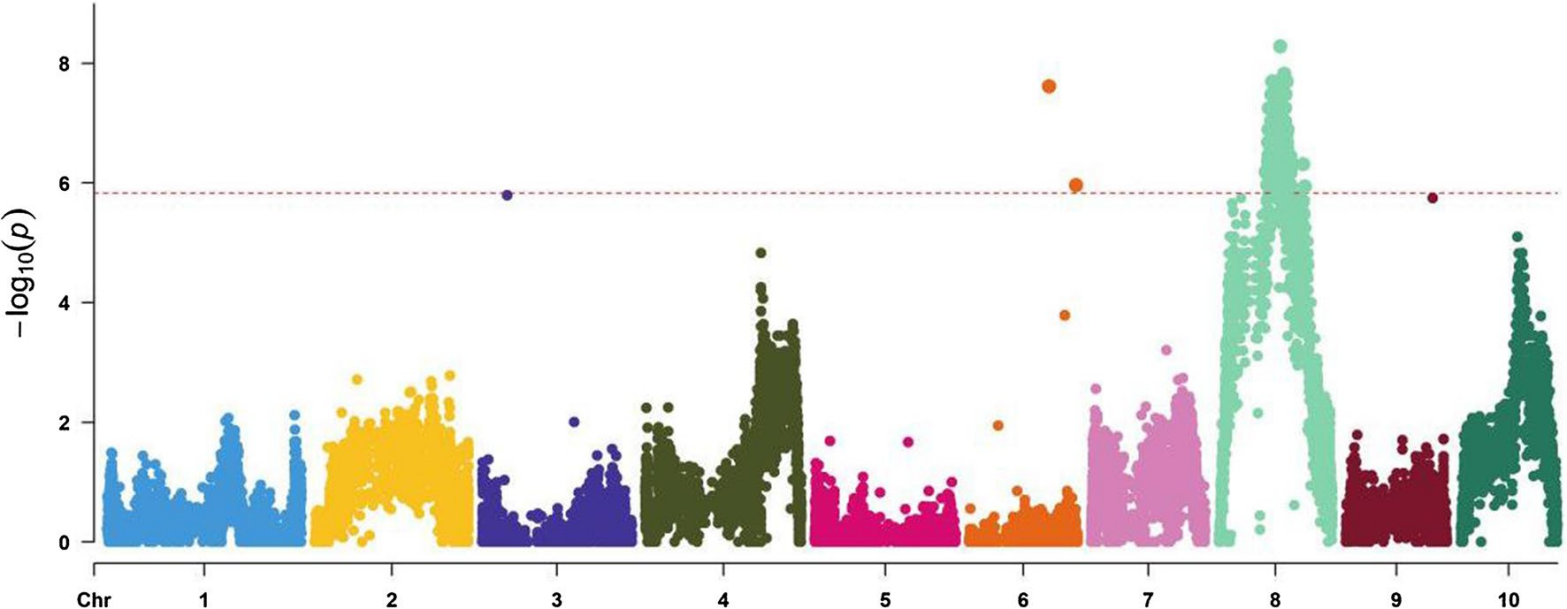
Selective genotyping analysis for tar spot complex (TSC) resistance in the DH population. *P* value means the difference between the allele frequencies of the two tails of the DH population, the horizontal line represents the threshold *P* value = 1.46 × 10^–6^

The selective genotyping results are shown in Fig. 3 and Table S3. A total of 298 SNPs distributed on two chromosomes showed a significant correlation between genotype and TSC resistance. Two SNPs S6_123687641 (*P* = 2.44 × 10^–8^) and S6_165635560 (*P* = 1.10 × 10^–6^) were located on chromosome 6 (bins 6.05 and 6.07). The rest of 296 SNPs were located on chromosome 8 (bins 8.03–8.04) and ranged from 72,383,502 bp to 111,193,065 bp with an interval of 38.81 Mb on B73 RefGen_v2 reference genome. The locus on chromosome 8, designated *qRtsc8-1* (Mahuku et al. 2016), had the lowest *P* value hence validating the GWAS results. The source of all TSC resistance alleles detected by selected genotyping was the resistant inbred CML495.

### Fine mapping of the major QTL *qRtsc8-1*

**Fig. 4:**
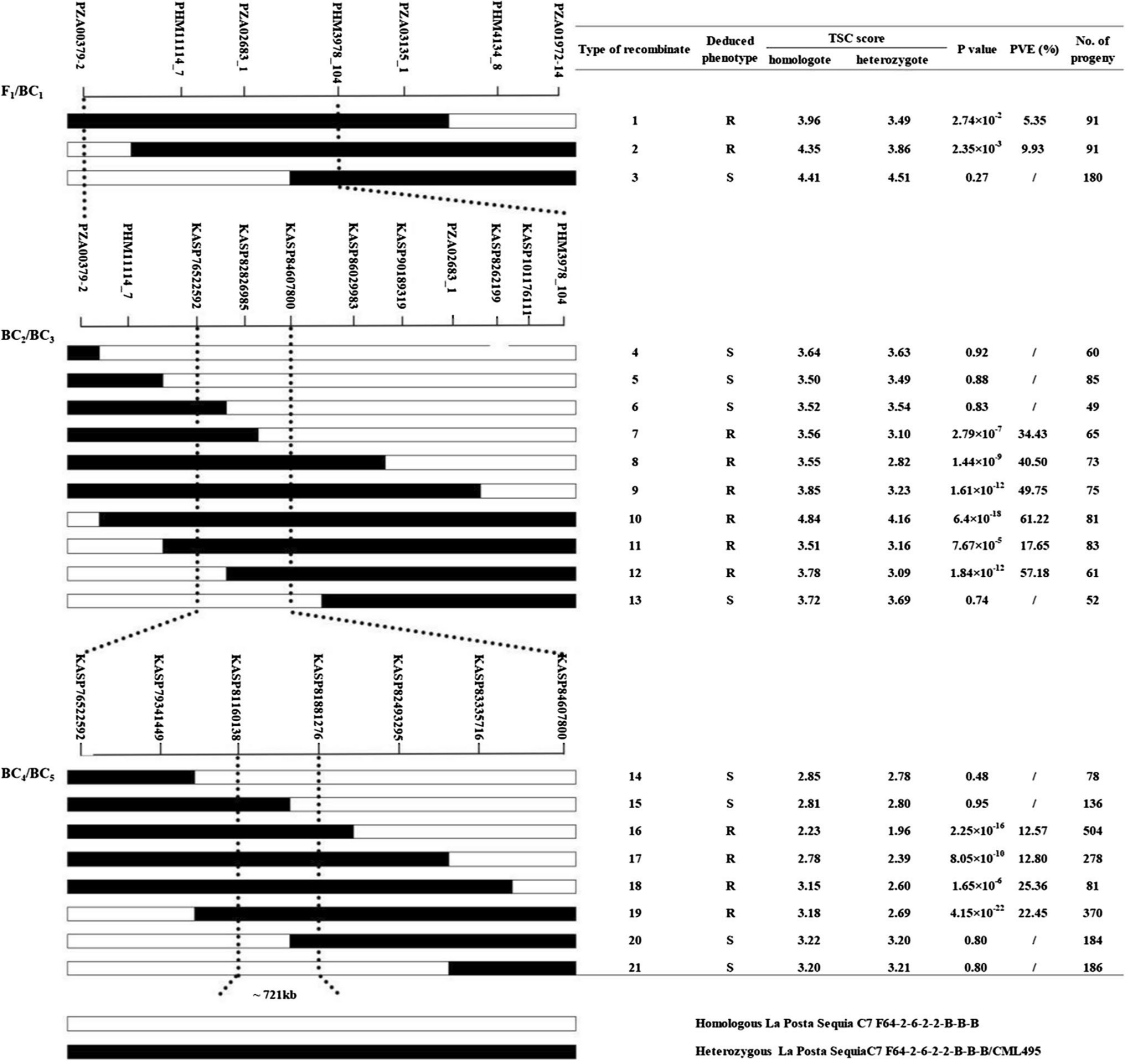
Fine mapping of *qRtsc8-1* with a progeny test strategy. The genetic structure of each recombinant type is shown in different colors on the left. White and black bars represent homozygous La Posta Sequia C7 F64-2-6-2-2-B-B-B genotype and heterozygous La Posta Sequia C7 F64-2-6-2-2-B-B-B/CML495 genotype, respectively. Significant differences (*P* < 0.05) in resistance to tar spot complex (TSC) between the two groups of genotypes indicate that *qRtsc8-1* is in the heterozygous region, and their corresponding recombinants are deduced as resistant (R). No significant differences (*P* ≥ 0.05) in resistance to TSC between the two groups of genotypes indicate that *qRtsc8-1* is not in the heterozygous region, and their corresponding recombinants are deduced as susceptible (S). The QTL *qRtsc8-1* was finely mapped between markers KASP81160138 and KASP81881276 within a  ~ 721 kb interval

To narrow down the region of *qRtsc8-1*, two flanking markers PZA00379_2 and PZA01972_14 were used to identify recombinants from 20 different crosses between resistant DH lines and susceptible DH lines. The recombinants were further genotyped with five KASP markers (PHM11114_7, PZA02683_1, PHM3978_104, PZA03135_1, and PHM4134_8) (Table S1) within the *qRtsc8-1* region. Three types of recombinants (R1 to R3) were detected and backcrossed to corresponding susceptible DH lines to generate BC_1_ progenies for fine mapping (Fig. 4). In the winter season from January to May in 2014, all 362 BC_1_ progenies were individually scored for TSC resistance and genotyped with all the seven KASP markers previously described. A two-way ANOVA was performed for the progeny testing to compare TSC scores between the two classes of genotype: homozygous La Posta Sequia C7 F64-2-6-2-2-B-B-B and heterozygous La Posta Sequia C7 F64-2-6-2-2-B-B-B/CML495. Resistance to TSC was significantly different between the two classes of genotype in recombinant types R1 and R2, indicating that the CML495 donor region harbored the resistance QTL of *qRtsc8-1*. There was no significant difference in resistance to TSC between the two classes of genotypes in recombinant type R3, indicating that the CML495 donor region did not harbor *qRtsc8-1*. QTL analysis of the recombinants narrowed down the *qRtsc8-1* to the region between markers PZA00379_2 and PHM3978_104 with a physical distance of ~ 33.80 Mb. The *qRtsc8-1* explained 5.35–9.93% of the total phenotypic variation based on the analysis on recombinants R1 and R2.

To further finely map *qRtsc8-1*, additional recombinant events were identified. A total of 512 plants from BC_2_ populations were planted in the summer season of 2014 and genotyped with two flanking markers PZA00379_2 and PHM3978_104 to identify recombinations, which were further investigated with nine KASP markers (PHM11114_7, KASP76522592, KASP82826985, KASP84607800, KASP86029983, KASP90189319, PZA02683_1, KASP8262199, and KASP101176111) (Table S1). Ten new types of recombinants (R4 to R13) were identified and backcrossed to susceptible DH lines to produce 684 BC_3_ progenies (Fig. 4). In the winter season from January to May in 2015, all the progenies were individually phenotyped and genotyped. The same progeny testing was conducted and narrowed down *qRtsc8-1* to an interval of ~ 8.09 Mb between markers KASP76522592 and KASP84607800. Recombinant types R6 and R12 confirmed the left boundary of the fine mapping region, and recombinant type R7 confirmed the right boundary. The phenotypic variation explained (PVE) value of *qRtsc8-1* ranged from 17.65 to 61.22% based on recombinants R7-R12.

Eight new types of recombinants (R14 to R21) were detected with seven KASP markers (KASP76522592, KASP79341449, KASP81160138, KASP81881276, KASP82493295, KASP83335716, KASP84607800) (Table S1) in BC_4_ populations (3515 plants) and backcrossed to susceptible DH lines to produce BC_5_ progenies. In the winter season from January to May in 2016, all the 1817 BC_5_ progenies were evaluated for resistance to TSC and genotyped using the seven markers (Fig. 4). Recombinant types R15 and R20 were deduced as susceptible by the progeny testing, indicating that *qRtsc8-1* was downstream of KASP81160138 and upstream of KASP81881276. Finally, *qRtsc8-1* was mapped to a physical distance of 721.14 kb between markers KASP81160138 and KASP81881276. The PVE value of *qRtsc8-1* detected in recombinants R16 to R19 ranged from 12.57 to 25.36%. The PVE value variations of *qRtsc8-1* between different recombinants and generations are due to the different genetic backgrounds and disease pressure variations in different evaluation seasons.

### Identification of candidate genes in the fine mapping interval of *qRtsc8-1*

Based on the annotation information of maize B73 reference genome obtained from maizeGDB, five genes including three putative uncharacterized proteins and two genes with known predicted function were identified within the fine mapping interval of *qRtsc8-1* (Table S4). GRMZM2G071228, GRMZM5G879762, and *GRMZM5G869967* are putative uncharacterized proteins, and their functions are still unknown. *GRMZM2G063511* encodes an integral membrane protein-like, that harbors the most significant SNP S8_81160155 detected by GWAS. *GRMZM2G073884* encodes a leucine-rich repeat receptor-like protein kinases (LRR-RLKs). Both *GRMZM2G063511* and *GRMZM2G073884* may play important roles in disease resistance.

Genetic variation analysis of *GRMZM2G063511* revealed that 326 polymorphic SNPs, 32 insertions, and 45 deletions were observed between the two parental lines. For *GRMZM2G073884*, 172 polymorphic SNPs, 20 insertions, and 21 deletions were observed between the two parental lines. Comparing CML495 with La Posta Sequia C7 F64-2-6-2-2-B-B-B, a ‘T’ deletion in the TSC resistant line CML495 showed a significant effect impact on protein-coding changes. The ‘T’ deletion causing a frameshift variation was located at position 519 bp in the coding region of *GRMZM2G063511*, and it has the potential to be used for the implementation of MAS in breeding lines.

### Validation of the production markers in breeding lines

Before deploying MAS, genotyping assays flanking the fine mapping interval need to be validated in breeding lines. In total, 8 of the 10 genotyping assays passed the technical validation process in the 471 breeding lines (Table 2). Two genotyping assays, including KASP81881276, did not pass the technical validation process, due to the low SNP calling success rates in the breeding lines (data not shown).

**Table 2:** Stepwise regression analysis of the eight KASP (Kompetitive Allele Specific PCR) markers in the *qRtsc8-1* fine mapping interval in the 471 breeding lines

KASP markers^a^	Favorable allele			Unfavorable allele			Full model *P *value^b^
	Allele	Frequency	TSC score	Allele	Frequency	TSC score	
KASP81160138	T	0.39	2.12	C	0.61	2.63	0.7918
KASP81160155	A	0.34	2.09	C	0.66	2.65	0.0036**
KASP81247441	A	0.71	2.35	G	0.29	2.66	0.8960
KASP81247607	G	0.64	2.32	A	0.36	2.65	0.5104
KASP81247664	A	0.59	2.32	G	0.41	2.61	0.8837
KASP81639091	G	0.67	2.28	A	0.33	2.64	0.9894
KASP81881034	T	0.83	2.10	C	0.17	2.59	0.9270
KASP82060813	G	0.80	2.40	A	0.20	2.48	0.4387

^a^KASP marker name, KASP followed by the position on chromosome 8, for example, KASP81160138 represents that the marker is located at the physical position 81,160,138 bp on chromosome 8, position corresponds to maize B73 RefGen_v2 reference genome

^b^** *P* < 0.01

The stepwise regression analysis result of the eight genotyping assays obtained from the technical validation process is shown in Table 2. The KASP81160155 with a full model *P* value of 0.0036 was identified as the most important production marker, explaining 23.07% of phenotypic variance. This SNP contained two alleles, “A” and “C”, in the breeding lines. The favorable allele in breeding lines was “A” with a MAF of 0.34. The average TSC scores of the breeding lines carrying the alleles “A” and “C” were 2.09 and 2.65, respectively. The favorable allele of “A” improved the TSC resistance by 15.02% compared to the average TSC score.

**Fig. 5:**
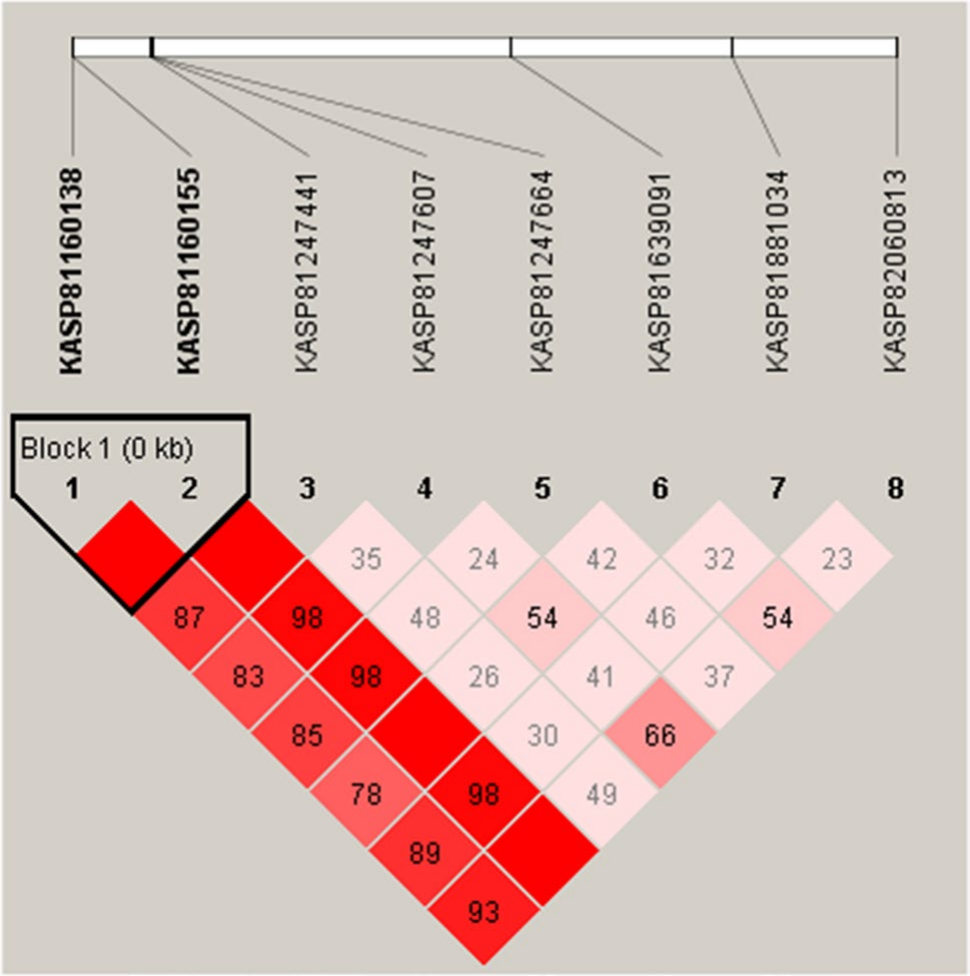
Linkage Disequilibrium (LD) heatmap and haplotype blocks across the eight KASP (Kompetitive Allele Specific PCR) markers in the *qRtsc8-1* region in 471 breeding lines. LD is measured as D’, ranging from 0 to 1. D’ value equals 1 is depicted in red (values not shown in the box) and less than 1 is depicted in shades of pink/light red. These values are D’ times 100

The LD analysis of the eight markers in 471 breeding lines revealed that KASP81160138 was located in the same haplotype block with KASP81160155 (Fig. 5). The favorable allele of “T” of KASP81160138 improved the TSC resistance by 12.82% compared to the average TSC score of all the breeding lines, which was a minor allele with a MAF of 0.39. Both KASP81160138 and KASP81160155 markers were located in *GRMZM2G063511*. Three possible haplotypes involving the two markers, “TA”, “TC”, and “CC”, were identified in all the breeding lines (Table 3). The haplotype H1 (“TA”) had a frequency of 0.36, showing the highest effect on improving TSC resistance. It improved the TSC resistance by 13.95% compared to the average TSC score across all the breeding lines. The remaining two haplotypes H2 (“TC”) and H3 (“CC”) reduced the TSC resistance by 7.93% and 8.90% compared to the average TSC score across all the breeding lines. The frequency of haplotype H2 was only 0.02. These two markers, KASP81160138 and KASP81160155, were identified as the production markers in breeding lines, which can be used for routine deployment of MAS to enrich the favorable alleles of *qRtsc8-1* in tropical maize breeding populations.

**Table 3:** Haplotype analysis results on two production markers, KASP81160138 and KASP81160155, in the *qRtsc8-1* fine mapping interval in 471 breeding lines

Haplotype ID	KASP81160138 allele	KASP81160155 allele	TSC score	Frequency	Contribution to TSC (%)^a^
H1	T	A	2.09	0.36	13.95
H2	T	C	2.64	0.02	−7.93
H3	C	C	2.62	0.62	−8.90

^a^The contribution of each haplotype was calculated by comparing the average tar spot complex (TSC) score of this haplotype with the average TSC score of all 471 breeding lines. The haplotype having the positive value of contribution to TSC can improve the resistance, while the haplotype having the negative value of contribution to TSC can reduce the resistance

## Discussion

Since 1904, TSC has been a major foliar disease of maize causing severe yield loss in many Central and Latin American countries. In 2015, tar spot was first detected in the U.S. (Illinois and Indiana) and has since spread across the midwestern US Corn Belt as well as southwest Ontario, Canada (Rocco da Silva et al. 2021). Development and deployment of maize varieties with genetic resistance is the most effective and sustainable approach to reduce yield losses caused by TSC. In the present study, GWAS and selective genotyping were used to detect and validate QTL conferring TSC resistance in maize. A total of 115 SNPs associated with TSC resistance were identified by GWAS in bin 8.03. The major QTL of *qRtsc8-1* explaining 14.97% of the phenotypic variance was validated by selective genotyping. The physical position of *qRtsc8-1* detected by GWAS and selective genotyping was between 73,734,307 bp to 90,835,374 bp and 72,383,502 bp to 111,193,065 bp, respectively. GWAS showed a higher mapping resolution. Two minor QTL in bins 6.05 and 6.07 were also detected by selective genotyping. These results revealed that the resistance to TSC in maize is controlled by a major QTL on chromosome 8 coupled with several minor QTL. The major QTL of *qRtsc8-1* was previously detected by Cao et al. (2017) and Mahuku et al. (2016). It shows that the QTL of *qRtsc8-1* is stable across different genetic backgrounds and environments. Moreover, this major QTL of *qRtsc8-1* was further verified with a fine mapping strategy and validated using production markers in breeding lines. The fine mapping results and the production of markers developed by the present study will facilitate MAS for TSC improvement.

High-density markers in the fine mapping region are essential to narrowing down the QTL region (Ren et al. 2017). Single sequence repeat (SSR), cleaved amplified polymorphic sequences (CAPSs), and insertion-deletions (InDels) have been widely used in QTL fine mapping in maize. However, they are laborious and time consuming. KASP is a homogeneous, fluorescence based single-step SNP genotyping assay, which has the characteristics of high throughput, high accuracy, low cost, and breeder friendliness (Semagn et al. 2014). It provides flexibility in genotyping thousands of samples with a few SNPs, such as fine mapping and MAS. Compared with SSRs, CAPSs, and InDels, KASP assays are more powerful and efficient for fine mapping and MAS (Fu et al. 2019). It has been developed for QTL fine mapping and MAS in many crops (Nair et al. 2015; Steele et al. 2018; Fu et al. 2019). In maize, KASP markers were developed for fine mapping of the major QTL *Msv1* and used for MAS in breeding programs developing MSV resistance (Nair et al. 2015). In the present study, with the availability of the GBS dataset of CML495 and La Posta Sequia C7 F64-2-6-2-2-B-B-B, the development of KASP markers in the fine mapping region has become feasible. A total of 19 new KASP markers were developed to saturate the *qRtsc8-1* region and identify key recombinations in the fine mapping populations. The KASP markers have proven to be high throughput, accurate and low cost for recombinant screening.

TSC resistance is a complex trait affected by both environmental and genetic factors. Obtaining accurate phenotypic data of each recombinant is crucial. A recombinant-derived progeny test has been widely used for fine mapping the major QTL conferring the resistance of several major maize diseases, either in self-pollinated progeny (Tao et al. 2013; Lv et al. 2021), or in backcrossed progeny (Yang et al. 2010; Deng et al. 2020). Progeny generated by self-pollination can evaluate the effect of all three genotypes and the type of gene action (Tao et al. 2013). Compared with self-pollinated progeny, the backcrossed progeny based on repeated backcrossing can reduce the genetic background noise and improve the reliability and accuracy of the phenotype (Yang et al. 2010). In the present study, the progeny test was performed in backcrossed progenies which were divided into two groups of genotype: heterozygous La Posta Sequia C7 F64-2-6-2-2-B-B-B/CML495 and homozygous La Posta Sequia C7 F64-2-6-2-2-B-B-B at the *qRtsc8-1* locus. Significant differences in resistance to TSC between the two groups of genotypes suggested that the QTL *qRtsc8-1* acts in a dominant manner. The production markers in the fine mapping interval of QTL *qRtsc8-1* were further validated in breeding lines, carrying two groups of genotype at the *qRtsc8-1* locus: homozygous CML495, and homozygous La Posta Sequia C7 F64-2-6-2-2-B-B-B. This result revealed that *qRtsc8-1* also acts in an additive manner.

The *qRtsc8-1* was narrowed down to a region of ~ 721 kb (B73 RefGen_v2). Candidate gene analysis revealed that two genes, *GRMZM2G063511* and *GRMZM2G073884*, are the putative candidate genes for *qRtsc8-1*. *GRMZM2G073884* encodes a LRR-RLKs, which plays an important role in plant growth and development and basal defense responses (Tang et al. 2010; Wang et al. 2019). LRR domains have long been considered to be related to plant disease resistance. *StLRTK1* encoding a LRR-RLKs in potato may participate in the disease resistance of *Phytophthora* (Wu et al. 2009). *GRMZM2G063511* encodes an integral membrane protein-like, which is a type of plasma membrane. The plasma membrane is involved in diverse physiological functions, like growth and development, signal transduction, ion transport, metabolism, and disease resistance (Hong et al. 2008; Yadeta et al. 2013). Genetic variation analysis revealed a frameshift variation in the T003 transcript of *GRMZM2G063511* between CML495 and La Posta Sequia C7 F64-2-6-2-2-B-B-B. Frameshift variation leads to the encoding of different amino acids after the 172 amino acid. Sanger sequencing is required to verify the variation. More studies are required to clone the gene accountable to TSC resistance in this fine mapping interval and to understand the molecular mechanisms underlying TSC resistance.

The development of markers tightly linked to *qRtsc8-1* is essential for deploying MAS for improving TSC resistance. Two production markers KASP81160138 and KASP81160155 were verified in breeding lines. The favorable allele of both makers was a minor allele, indicating that increasing the frequency of the favorable allele in breeding programs is valuable. The two production markers can be used to enrich the favorable allele of *qRtsc8-1* in early generations (F_2_, F_3_, BC_1_, BC_2_), which allows breeders to focus on fewer lines in subsequent generations. Since CIMMYT has already developed and routinely employs a large number of disease markers for MAS, such as MSV, maize lethal necrosis, and maize aflatoxin, the two markers reported here can be used together with those markers to develop maize germplasm with multiple disease resistance.

Genomic selection, an extension of MAS, has been reported to improve TSC resistance effectively by Cao et al. (2017, 2021), where moderate to high prediction accuracies were obtained in different populations. Genomic prediction analysis with significantly associated markers has the potential to improve prediction accuracy (Cao et al. 2021). Incorporating the production markers of KASP81160138 and KASP81160155 into genomic prediction has the potential to improve TSC resistance in breeding programs.

Accurate phenotypic evaluation with reliable artificial inoculation methods is crucial for genetic dissection of the TSC resistance and development of TSC resistant varieties. However, artificial inoculation methods are still not available. First, the main player *P. maydis* is biotrophic and therefore cannot be cultured for mass production in the laboratory. Furthermore, the complexity of the interaction between the three TSC causal pathogens is not well understood. In the present study, all the results are based on phenotypic data obtained from multiple environment trials under natural screening conditions. Reliable artificial inoculation methods need to be explored in further research.

## Supplementary Information

Below is the link to the electronic supplementary material.

Supplementary file1 (DOCX 22 kb)

## Data Availability

The datasets generated during and/or analyzed during the current study are available from the corresponding author on reasonable request. Supplementary data associated with this article can be found in the online version.
