# Supplementary material for: Identification and fine mapping of a major QTL (qRtsc8-1) conferring resistance to maize tar spot complex and validation of production markers in breeding lines
Source: Theor Appl Genet. 2022 Feb 18;135(5):1551–63. doi: 10.1007/s00122-022-04053-8 (PMC9110495; doi:10.1007/s00122-022-04053-8)
Supplement: Supplementary file 1 — Supplementary file1 (DOCX 22 kb) [file 122_2022_4053_MOESM1_ESM.docx]

**Table S1** The list of KASP markers for fine mapping and validation of *qRtsc8-1*

| KASP markers^a^ | Chromosome | V2 position^b^ | V4 position^c^ | Sequence |
| --- | --- | --- | --- | --- |
| PZA00379_2 | 8 | 67381250 | 68445388 | TTCCGCCAGCATGGGCGCAACATCCTTGTCGCTTGCAGAGCCTACCTGGA[C/T]GGTGCCCAGGTTGGATGCCTATCKGGCGATGGAGTGCAGGAYGTTGATGA |
| PHM11114_7 | 8 | 72628174 | 73809393 | CTTGTCAGACCGATCGTCATGTTCTTTCTTGCCTCCAACCGAAACCGGCA[A/G]GTCTGACATGTCTAGCTTCTGATACGCAGGGTCGCCGTCCTGGTTGCGCC |
| KASP76522592 | 8 | 76522592 | 77933751 | TGCAGTGATGAGTACAAATGTTGTTTTGCTGCCTGGATACAGTACAAATG[T/C]TGTTTCCATTGATCAGAGGCCTCAACGGCATTTTCTTTTTATTAGCAAAC |
| KASP79341449 | 8 | 79341449 | 80751963 | CCTACATGGCACGACGTCAGTGGTGAGCAGCGCCGAGGCCTAGCCAAGCG[G/A]AGAACCCGCTCAAGAAGCGGTGGCAGCCATGCCCACCTCGCTCACCACGT |
| KASP81160138* | 8 | 81160138 | 82664864 | CGCGGTTTGCTGATCAAAATGGGGTCTTTCTCGGCTGCAGGTGCACCGGG[C/T]CGCGTGATTCCGGCGGCTTGATTTGGAATTTGTGATCTTTCGGGGCATTC |
| KASP81160155* | 8 | 81160155 | 82664881 | AATGGGGTCTTTCTCGGCTGCAGGTGCACCGGGCCGCGTGATTCCGGCGG[C/A]TTGATTTGGAATTTGTGATCTTTCGGGGCATTCGTCGGTTTGTCTGTCGG |
| KASP81247441* | 8 | 81247441 | 82754895 | GACAAGGGAGGAGCTGCTTCCAAGTGAAGCTGGAATGCTGCCCGTCAGAT[A/G]GTTACCTGAAAGGCGTAAATCCGAGAGGTTGCCGAGGAGGCTCCCCAGAC |
| KASP81247607* | 8 | 81247607 | 82755061 | GAGACAGTCTCCGGGATCTCGCCGCTGATAAAATTGTTGGAGCTGAGGTT[A/G]ATGTACCGCAGCCGGTTTAACTGGCCAAGCTCAGCCGGGATCTGACCCTG |
| KASP81247664* | 8 | 81247664 | 82755118 | CGCAGCCGGTTTAACTGGCCAAGCTCAGCCGGGATCTGACCCTGCAGCTG[G/A]TTGTTTGGCATGTGGATCCTTGTGAGGAACGTGAGGTTGCCGATGCAAGG |
| KASP81639091* | 8 | 81639091 | 83224235 | GCTTTGTTTTTAAGACCACAAAGGAGACTCGCGTCAAACTTCCAGCTGCC[G/A]GCCAGATTTCTCACAAGCTCCCTGAGGTCAAGAGTTCAGTTGCAGCTGCA |
| KASP81881034* | 8 | 81881034 | 83444848 | CCTGCTGGTCTTGCCGCCGACGACCGTGGCAAGCGCGTTCCTGACGGCCA[C/T]CGCGACGCCACACCCCCTGACCACCCGCCTCTCGCAGCACTCACGCTCGG |
| KASP81881276 | 8 | 81881276 | 83444606 | TGTTGGAACTCGAGGAAGACCAGGAGGAGGAGGAGGAGGAGCACGGCGGC[A/G]TCGCCTGGCTGCCGCCGGGCCTCCGGAGGAGGGTGATGCGGGGGAGCACG |
| KASP82060813* | 8 | 82060813 | 83647465 | AGCCCCTCGGCAGCGCTAATACTGGACTACTCGAACCTTTGACCTAACGT[G/A]CCAAGCTTCGATCCGGAAGTAGCTACATGTAGCGGAGAGAAAAGCAAAGT |
| KASP82493295 | 8 | 82493295 | 84079217 | ATCGGGCATGGGAATCTGTGCGTGATTTGGTTGCGCGGCTTAATTCGTGC[A/G]GCAGCATGTGTGCGTGCGCATTTGTTTTCTGTGCCCGATGGTGAGTTGCT |
| KASP82826985 | 8 | 82826985 | 84424240 | TTGGTGAGGAGGAGAGGAGGGGAGGAAAGGAGGTGTTGCGGATGGGCGTC[G/A]GTGCGGAGCCCAAGGAGCAGGCGGCGTCCGCCGCAGCTCTGGCGGTGGAG |
| KASP83335716 | 8 | 83335716 | 84947827 | CTACATATGACAGCCCAATAGCCCACTAACTCAAAACCCTAGCAGCAGCC[C/A]CTGAAGTCGAATCCCCTCACACTATAAAGCTCCTCCATTGCGCCGCCGCC |
| KASP84607800 | 8 | 84607800 | 86257738 | GCACACTTACTAGTTTGCTCTTTTTATGTTTTCTAGGCCTGCTTTGTTTT[C/T]ATATTATATTGTTTGCTCTGTTCATTGTTTGCTGCTTTATTTTCCCGTGC |
| KASP86029983 | 8 | 86029983 | 87693625 | CGCCGCGCTGAGCTCCTCCTCTCACTGCTGCTCCTCCCACACGCTCTCCC[A/C]TTGTTGCTAGTGCCGCCGCTCGTCATTGCTGCCAAGCGCTATTGCTTGCA |
| KASP90189319 | 8 | 90189319 | 92079862 | CCCGGCACCCCCCGCGGCAGCGGCGCGCAGAAGACCCCAGTGTAGTTGCA[G/C]ACCCCCGGGCCCACCCAGTCGGCGGTGAAGCCCCTGGGGTCGGAGAAGAT |
| PZA02683_1 | 8 | 91993060 | 93781941 | CGMTGTGCGCTTGGCGAACGCGGCCACCTGCATGTTCAGCACGGCCAAAT[G/T]GTCGATGACCGGGTACTTGGATGCATGGCGCCCGGCYGTGGMCGTGCAAA |
| KASP98262199 | 8 | 98262199 | 100158104 | GCAACCGAGCTTCCCCCGCAGCCCCTCAACATCGACGTGTCATAGGTGTT[T/C]GACGGAATGCTCGTGGGAAATATTATGTTGTACGCAGCAGCCCCGACGTC |
| KASP101176111 | 8 | 101176111 | 103219737 | AGTCCAGAGATTCCCTGCAATCGCTCGGCTTTTGGACTAGCGGGGTCGTG[G/A]ATGCCCGGAGCTGCAGAGGCCACTTGCGTCCGTCCGATCTGCCCAGTGGA |
| PHM3978_104 | 8 | 101178480 | 103222106 | GGGATCGATCGACTTGCAGCGTCCATAGAGTATGTCCTTGCGAGCGAATG[A/G]GCAGCACGATTGACTTGGGCATAGTAGAGTAGACACTAGACAGGTAAAAA |
| PZA03135_1 | 8 | 101775361 | 103881572 | CAGCAGACCCGCCGCGCTGGGCAGCGACCGCCAGCACCCTTCCCCGTGCG[A/C]CCGGATGTACGCCACCAGCCGCTCGTCCTCCTCCTTGGTCCACGCGCCCT |
| PHM4134_8 | 8 | 107094242 | 109549008 | TTGGACAAGCCCAACATACCAAAGCCTCCACCAGAGACGGAGAGGCTAGT[C/G]GTTATGAGGGGCGACTACAGCAAAATGGACACATATTATGTCATGCCCAA |

^a^ KASP markers named starting with PZA or PHM were obtained from the Maize KASP Assays in this study; KASP markers named starting with KASP were newly developed markers; *markers used for validation in the 471 breeding lines

^b^ Position corresponds to maize B73 RefGen_v2 reference genome

^c^ Position corresponds to maize B73 RefGen_v4 reference genome

**Table S2** The top twenty significantly associated SNPs for tar spot complex (TSC) resistance revealed by GWAS analysis on chromosome 8

| SNP^a^ | V4 position^b^ | *P*-value | Allele^c^ | MAF^d^ | PVE(%)^e^ |
| --- | --- | --- | --- | --- | --- |
| S8_79341449 | 80751963 | 2.89×10^-17^ | A/G | 0.26 | 12.90 |
| S8_79424352 | 80843411 | 3.85×10^-17^ | A/T | 0.26 | 13.48 |
| S8_79424520 | 80843579 | 8.11×10^-17^ | C/T | 0.26 | 13.34 |
| S8_80045991 | 81588028 | 8.84×10^-17^ | G/T | 0.26 | 12.50 |
| S8_80387047 | 81983328 | 1.56×10^-17^ | C/T | 0.27 | 14.00 |
| S8_80387056 | 81983337 | 1.56×10^-17^ | G/T | 0.27 | 14.00 |
| S8_80495607 | 82095436 | 2.02×10^-17^ | C/G | 0.26 | 13.60 |
| S8_80792155 | 82400012 | 3.36×10^-17^ | C/A | 0.26 | 13.08 |
| S8_80794351 | 82402208 | 8.74×10^-17^ | T/A | 0.31 | 14.97 |
| S8_81160138 | 82664864 | 1.70×10^-17^ | C/T | 0.26 | 13.08 |
| S8_81160139 | 82664865 | 1.37×10^-17^ | C/T | 0.26 | 13.15 |
| S8_81160155 | 82664881 | 4.36×10^-18^ | C/A | 0.26 | 13.57 |
| S8_81881034 | 83444848 | 1.38×10^-17^ | C/T | 0.27 | 13.40 |
| S8_81881083 | 83444799 | 1.48×10^-17^ | G/C | 0.26 | 13.22 |
| S8_81881084 | 83444798 | 1.48×10^-17^ | G/A | 0.26 | 13.22 |
| S8_82377096 | 83963039 | 4.19×10^-17^ | C/T | 0.27 | 12.76 |
| S8_82377154 | 83963097 | 1.29×10^-17^ | C/A | 0.27 | 13.17 |
| S8_82378764 | 83964707 | 5.52×10^-17^ | G/A | 0.27 | 13.05 |
| S8_82827016 | 84424271 | 5.66×10^-17^ | C/T | 0.27 | 12.83 |
| S8_82856104 | 84439248 | 6.88×10^-17^ | T/C | 0.26 | 14.60 |

^a^ SNP name, chromosome_position, for example, S8_79341449 represents that the SNP is on chromosome 8 and the physical position is 79,341,449 bp based on B73 RefGen_v2 reference genome

^b^ Position corresponds to maize B73 RefGen_v4 reference genome

^c^ major allele / minor allele

^d^ MAF, minor allele frequency

^e^ PVE, phenotypic variation explained

**Table S3** Significantly associated SNPs on chromosome 6 and the top ten significantly associated SNPs on chromosome 8 for tar spot complex (TSC) resistance detected by selective genotyping analysis in the DH population

| Chromosome | SNP^a^ | V4 position^b^ | Allele frequencies of CML495^c^ | | *χ^2^* | *P*-value |
| --- | --- | --- | --- | --- | --- | --- |
|  |  |  | In R group | In S group |  |  |
| 6 | S6_123687641 | 127574522 | 1.00 | 0.00 | 31.11 | 2.44×10^-8^ |
| 6 | S6_165635560 | 169787085 | 0.94 | 0.10 | 23.75 | 1.10×10^-6^ |
| 8 | S8_82377096 | 83963039 | 1.00 | 0.05 | 31.50 | 2.00×10^-8^ |
| 8 | S8_83335753 | 84947864 | 1.00 | 0.05 | 31.50 | 2.00×10^-8^ |
| 8 | S8_88047096 | 89844328 | 1.00 | 0.05 | 31.50 | 2.00×10^-8^ |
| 8 | S8_88275352 | 90095997 | 1.00 | 0.05 | 31.50 | 2.00×10^-8^ |
| 8 | S8_88438999 | 90224230 | 1.00 | 0.05 | 31.50 | 2.00×10^-8^ |
| 8 | S8_88813183 | 90589294 | 1.00 | 0.05 | 31.50 | 2.00×10^-8^ |
| 8 | S8_94872293 | 96678945 | 1.00 | 0.00 | 34.11 | 5.22×10^-9^ |
| 8 | S8_101118384 | 103152735 | 1.00 | 0.00 | 32.06 | 1.49×10^-8^ |
| 8 | S8_101118399 | 103152750 | 1.00 | 0.00 | 32.06 | 1.49×10^-8^ |
| 8 | S8_101118400 | 103152751 | 1.00 | 0.00 | 32.06 | 1.49×10^-8^ |

^a^ SNP name amd chromosome_position, for example: S6_123687641 represents that the SNP is on chromosome 6 and the physical position is 123687641 bp based on B73 RefGen_v2 reference genome

^b^ Position corresponds to maize B73 RefGen_v4 reference genome

^c^ R group, resistant group; S group, susceptible group

**Table S4** Candidate genes in the *qRtsc8-1* region

| Gene ID | V2 position^a^ | V4 position^b^ | Description/Annotation |
| --- | --- | --- | --- |
| GRMZM2G063511 | 81159824-81164543 | 82664581-82669224 | integral membrane protein like |
| GRMZM2G073884 | 81244522-81248033 | 82751665-82755501 | leucine-rich repeat receptor-like protein (LRR-RLKs) |
| GRMZM2G071228 | 81414251-81419501 | 82883180-82884110 | putative uncharacterized protein |
| GRMZM5G879762 | 81641236-81641401 | 82884315-82885231 | putative uncharacterized protein |
| GRMZM5G869967 | 81649770-81653765 | 83444248-83444925 | putative uncharacterized protein |

^a^ Position corresponds to maize B73 RefGen_v2 reference genome

^b^ Position corresponds to maize B73 RefGen_v4 reference genome
